# A Lightweight Model for Small-Target Pig Eye Detection in Automated Estrus Recognition

**DOI:** 10.3390/ani15081127

**Published:** 2025-04-13

**Authors:** Min Zhao, Yongpeng Duan, Tian Gao, Xue Gao, Guangying Hu, Riliang Cao, Zhenyu Liu

**Affiliations:** 1 College of Animal Science, Shanxi Agricultural University, Taigu 030801, China; 18734919105@163.com (M.Z.); awsl5214789630@163.com (T.G.); gaoxue202302@163.com (X.G.); huguangying1968@163.com (G.H.); 2 College of Information Science and Engineering, Shanxi Agricultural University, Taigu 030801, China; duan86998310@163.com; 3 College of Agricultural Engineering, Shanxi Agricultural University, Taigu 030801, China; 4 Dryland Farm Machinery Key Technology and Equipment Key Laboratory of Shanxi Province, Taigu 030801, China

**Keywords:** sow estrus, pig eyes, deep learning, animal welfare, ECA–YOLO

## Abstract

In modern large-scale pig farming, accurately identifying sow estrus and ensuring timely breeding are crucial for maximizing economic benefits. However, the short duration of estrus and the reliance on subjective human judgment pose significant challenges for precise insemination timing. To enable non-contact, automated estrus detection, this study proposes an improved algorithm, Enhanced Context-Attention YOLO (ECA-YOLO), based on YOLOv11. The model utilizes ocular appearance features—eye’s spirit, color, shape, and morphology—across different estrus stages as key indicators. The MSCA module enhances small-object detection efficiency, while the PPA and GAM modules improve feature extraction capabilities. Additionally, the Adaptive Threshold Focal Loss (ATFL) function increases the model’s sensitivity to hard-to-classify samples, enabling accurate estrus stage classification. The model was trained and validated on a dataset comprising 4461 images of sow eyes during estrus and was benchmarked against YOLOv5n, YOLOv7tiny, YOLOv8n, YOLOv10n, YOLOv11n, and Faster R-CNN. Experimental results demonstrate that ECA-YOLO achieves a mean average precision (mAP) of 93.2%, an F1-score of 88.0%, with 5.31M parameters, and FPS reaches 75.53 frames per second, exhibiting superior overall performance. The findings confirm the feasibility of using ocular features for estrus detection and highlight the potential of ECA-YOLO for real-time, accurate monitoring of sow estrus under complex farming conditions. This study lays the groundwork for automated estrus detection in intensive pig farming.

## Simple Summary

This study proposes ECA–YOLO, an improved YOLOv11-based algorithm, for automated estrus detection in pigs using non-contact ocular analysis. The model integrates enhanced context-aware mechanisms to address challenges like short estrus duration and reliance on human expertise, improving detection accuracy under complex farming conditions. By leveraging advanced attention modules and adaptive training strategies, it achieves real-time performance for continuous monitoring. This approach supports efficient reproductive management in intensive pig farming systems.

## 1. Introduction

Accurately determining the estrus period of sows in modern large-scale pig farming is crucial for optimizing reproductive management. This not only facilitates the precise scheduling of mating [1], reducing economic losses caused by mistimed or missed inseminations, but also effectively decreases non-productive days, thereby improving litter size and overall reproductive efficiency [2,3,4]. Consequently, accurate estrus detection significantly enhances the economic benefits of pig farms. Previous studies have classified the estrus cycle of sows into three distinct phases: early, mid, and late estrus [5]. Sows exhibit notable phenotypic and behavioral changes across these phases. For instance, the vulva undergoes significant alterations in color and size before and after estrus [6]. During the early estrus phase, sows tend to display restlessness and emit low-frequency, continuous grunting sounds [7]. At mid-estrus, the vulva becomes deep red, reaches peak swelling, and secretes semi-transparent viscous mucus [8].

Traditional estrus detection methods primarily rely on visual inspection and boar exposure tests, such as the back pressure test (BPT) [9], to determine estrus status. However, these approaches are labor-intensive and time-consuming, making them unsuitable for automated inspection in large-scale modern farms [10]. Consequently, numerous researchers have explored automated estrus detection techniques. Lei et al. [11] proposed an intelligent mobile monitoring method based on a bionic boar model, which simulates boar vocalization, scent, and tactile stimulation to detect estrus in weaned sows. Xu et al. [6] developed an automated estrus detection method utilizing LiDAR cameras to monitor vulvar swelling before and after estrus. Experimental results demonstrated a measurement error of 3.4 ± 3.0 mm, indicating high detection accuracy.

With the rapid development of deep learning and artificial intelligence, animal detection methods represented by machine vision have emerged as promising solutions due to their non-contact and cost-effective nature. Wu et al. [12] improved the YOLOv8s model and integrated it with a robotic patrol system to achieve cage egg localization and quality inspection, significantly enhancing detection accuracy. Xia and Luo proposed the hybrid YOLO model PAB-Mamba-YOLO [13] and the improved PBR-YOLO [14], respectively, for identifying aggressive behaviors, such as tail and ear biting in weaned piglets. Huang et al. [15]. developed IAT-YOLO, an enhanced YOLOv7 model for detecting black pigs in low-light environments. In the field of automated estrus detection, Wang et al. [16]. introduced YOLO-TransT to track estrous cows in natural scenes, achieving a tracking accuracy of 85.5%. Zheng et al. [17] proposed FD-YOLOV5s, a vulvar temperature detection model that extracts sow vulvar temperature from infrared thermal images to identify estrus status, laying a foundation for automated inspection. Wang et al. [18] developed an early warning model, MobileNetV3-ResNet, for sow estrus sound monitoring by extracting Log-mel spectrograms and feeding them into a CNN for classification. Chen et al. [19] proposed a CNN-based estrus sound recognition method, which was validated to be effective in real pig farms. Wang et al. [20] used LSTM to identify estrus in dairy cows based on vocal characteristics. These studies demonstrate that phenotypic and behavioral features can serve as reliable indicators for estrus detection.

Ocular diagnosis is a crucial component of traditional Chinese medicine (TCM) inspection, which assesses physiological health and disease risks by observing changes in the eye’s spirit, color, shape, and morphology. With advancements in artificial intelligence, integrating ocular diagnosis with AI enables the objective and visualized analysis of eye images through automated capture, recognition, and processing, facilitating health assessment [21,22]. To this end, Wang et al. [23] proposed a deep learning-based eye recognition framework, MiCore-Net, which achieved accuracy rates of 99.08% and 96.12% on the CASIA-Iris-IntervalV4 and UBIRIS datasets, respectively. Research indicates that porcine eyes, due to their morphological similarity to human eyes, serve as a common ex vivo model for vision science studies [24,25,26]. Theoretically, they could also provide an additional physiological indicator for detecting estrus in animals, complementing vulvar and vocalization-based assessments.

Current animal eye recognition research primarily focuses on iris and fundus identification. Li et al. [27] employed an automatic dual-imaging system to capture surface and fundus images of bovine eyes, using novel dynamic analysis of 29 ocular features to predict blood vitamin A levels. Rojas-Olivares et al. [28] obtained retinal images from 152 lambs and accurately identified their identities through retinal recognition. Saygili et al. [29] developed an image-based method utilizing retinal vascular patterns for cattle identification, achieving a maximum accuracy of 92.25%. Zhang et al. [30] targeted the eye region, extracting temperature data to detect abnormal body temperatures in pigs. Schmitz et al. [31] accurately distinguished different activity patterns in animals based on high-precision ocular morphological measurements. While these retina-based image recognition methods demonstrate high accuracy, they require specialized imaging techniques and extensive image processing, rendering them unsuitable for cost-effective, easy-to-operate, and deployable applications in livestock farming.

To explore the feasibility of using porcine eye characteristics (spirit, color, form, and state) for estrus detection and to develop a non-contact, lightweight, low-cost, and easily deployable AI-assisted ocular diagnosis method for continuous estrus monitoring, this study proposes an enhanced context-aware YOLO-based model, ECA–YOLO. The model accurately classifies sows into four estrus stages—pre-estrus, mid-estrus, post-estrus, and non-estrus—based on eye appearance features across different estrus phases. The key contributions of this study are as follows:

(1) A comprehensive analysis of porcine eye features across different estrus stages, leading to the establishment of a dataset encompassing eye images of sows in pre-estrus, mid-estrus, and post-estrus phases;
(2) Algorithmic improvements, including the introduction of the MSCA module to enhance small-object detection efficiency, the PPA and GAM modules to strengthen feature extraction capabilities, and the adaptive threshold focal loss (ATFL) function to improve model focus on hard-to-classify samples;
(3) A comparative analysis of ECA–YOLO against YOLOv5n, YOLOv7tiny, YOLOv8n, YOLOv10n, YOLOv11n, and Faster R-CNN using the estrus sow eye dataset.

## 2. Materials and Methods

### 2.1. Materials

#### 2.1.1. Data Collection

**Figure 1:**
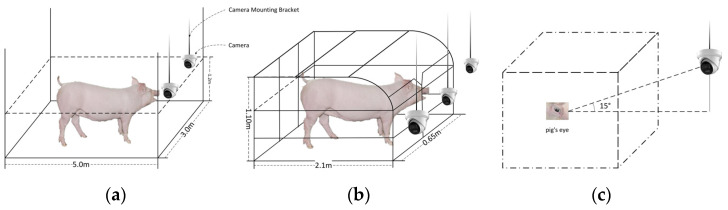
Schematic of data acquisition process. (**a**) Group housing, (**b**) Sow positioning pen, (**c**) Distribution of camera and pig eye angle.

The research team conducted data collection at a pig farm in Xiashan Village, Yong’an Town, Fenxi County, Shanxi Province, China (longitude: 111.603493, latitude: 36.649719). The study involved 25 Yorkshire pigs (Large White, Latin name: Yorkshire), aged 6–7 months, including 10 sows housed in group pens and the remainder in individual gestation stalls. To minimize experimental variability, all sows were completely isolated from boars throughout the study. Physiological health monitoring and data collection were carried out continuously over a three-month period (June to September 2023) to ensure comprehensive and accurate estrous cycle recording. The data collection process is illustrated in Figure 1. The selection of 6–7-month-old gilts was based on their fully developed reproductive organs and functional maturity, marking the onset of sexual maturity. Data acquisition was performed using a Hikvision DS-2CD3346WDV3-I camera. A total of 159 valid video clips, each lasting between 5 and 120 s at 30 fps, were recorded. The videos captured eye images of sows across four estrous stages: anestrus, proestrus, estrus, and post-estrus. Frame extraction techniques were applied, yielding 2994 images for further analysis.

#### 2.1.2. Data Set Construction

The estrous cycle in sows is a complex physiological process characterized by significant dynamic fluctuations in hormone levels [32]. The concentrations of estradiol (E2), progesterone (P4), and luteinizing hormone (LH) serve as key endocrine indicators for assessing estrus status. The periodic fluctuations of these hormones directly reflect different estrus phases, providing a biological foundation for accurate estrus identification. During data collection, periodic blood samples were obtained from experimental sows, and serum concentrations of E2, P4, and LH were measured using radioimmunoassay (RIA) [33] as a reference for estrous phase classification: in the pre-estrus phase, E2 gradually increases, while P4 and LH remain at baseline levels; during mid-estrus, E2 peaks, accompanied by a rapid surge in LH; in the late-estrus phase, E2 declines, P4 rises progressively, and LH returns to baseline. This hormone fluctuation-based calibration method provides an objective standard for estrus phase labeling within the dataset.

Previous studies by our research team have demonstrated that hormonal fluctuations around estrus are accompanied by a series of physiological changes, which manifest in behavioral and phenotypic traits, such as vulvar swelling and reddening, ear erection, and the standing reflex. In terms of ocular characteristics, sows exhibit distinct eye appearance changes across estrus phases. During early estrus, they show signs of anxiety or restlessness, displaying heightened vigilance toward environmental changes or farm personnel. In mid-estrus, sows exhibit a standing reflex with wide-open, dilated eyes, a vacant gaze, and mild vascular dilation within the eye. As estrus concludes, the eyes appear slightly fatigued, with reduced attentiveness to the surroundings. In contrast, non-estrus sows exhibit no significant changes in eye size or morphology, maintaining a calm gaze without fixating on specific targets. These phenotypic variations provide a theoretical basis for estrus detection based on ocular features.

**Figure 2:**
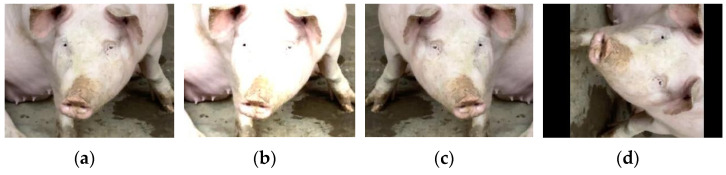
Data enhancement methods. (**a**) Original, (**b**) Brightness Increase, (**c**) Flip Image, (**d**) Angle of Rotation.

**Figure 3:**
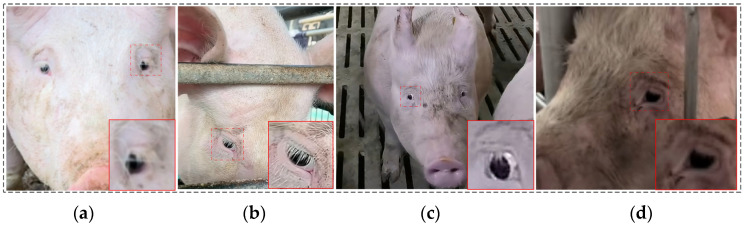
Partial image of the estrus sow eye dataset (eye features highlighted). (**a**) Non-estrus, (**b**) Pre-estrus, (**c**) Mid-estrus, (**d**) Late-estrus.

Two veterinary experts from the College of Animal Science and Veterinary Medicine, Shanxi Agricultural University, participated in data collection and annotation. One expert, specializing in hormonal regulation of the estrous cycle, assisted in blood sample collection, while the other, with over ten years of experience in pig farming management and behavioral phenotyping, was responsible for ocular annotation. Labeling was performed using the LabelImg software, with specific version and runtime details provided in the Experimental Platforms section. To mitigate excessive similarity between consecutive image frames, the structural similarity index (SSIM) [34] was employed to eliminate redundant images. For clarity and classification purposes, four labels were used to represent estrus stages in this study: Pre-estrus, Mid-estrus, Late-estrus, and Not-estrus. Following rigorous selection and filtering, a total of 1487 images were compiled into the estrus sow eye dataset, encompassing ocular images of sows across the four estrus stages. Data augmentation techniques were applied to expand the dataset threefold, resulting in 4461 images. The dataset was randomly split into training, testing, and validation sets at a ratio of 7:1:2, yielding 3122 training images, 446 testing images, and 893 validation images. Data augmentation methods and outcomes are illustrated in Figure 2, detailed dataset distribution is presented in Table 1, and partial images of the constructed estrus sow eye dataset are shown in Figure 3 (zoomed in to highlight eye appearance features at each stage).

Small objects in images refer to target objects that occupy a minimal portion of the image, typically characterized by small target size, low pixel occupancy, limited feature information, susceptibility to loss, and vulnerability to background interference. The definition of small objects can be classified into two approaches: relative size and absolute size. According to the SPIE definition, an object is considered small if its area is less than 80 pixels in a 256 × 256 image, which is approximately 0.12% of the total image area (relative size definition) [35]. In contrast, the COCO dataset, a widely used benchmark in object detection, defines small objects as those smaller than 32 × 32 pixels (absolute size definition). From the point of view of the target size of the detected eye, considering an input image size of 640 × 640 pixels, the average size of the sow’s eye region in our dataset is approximately 26 × 26 pixels, accounting for about 0.17% of the image area, classifying it as a small object.

### 2.2. Methods

#### 2.2.1. YOLOv11

YOLO11 was released in September 2024, building upon the success of previous YOLO versions, with new features and improvements to enhance performance and flexibility. Compared to its predecessors, YOLO11 introduces a redesigned backbone and neck architecture, replacing the C2f module from YOLOv8 with the C3k2 module and incorporating a novel C2PSA module in the backbone. These modifications enhance feature extraction capabilities while an optimized training pipeline improves processing speed without compromising accuracy. Additionally, YOLO11 features a more lightweight design, significantly improving computational efficiency. The YOLO11m variant achieved higher mean average precision (mAP) on the COCO dataset while using 22% fewer parameters than YOLOv8m, striking an optimal balance between accuracy and efficiency.

#### 2.2.2. MSCA Module

Due to the small proportion of the eye region within the entire image, feature extraction and representation become more challenging. Enhancing the model’s focus on small objects and improving contextual feature extraction are crucial for boosting the performance of estrus sow eye recognition. Multi-Scale Convolutional Attention (MSCA) [36] is a novel enhancement module designed for multi-scale contextual feature extraction, consisting of three components: depth-wise convolutions for local information aggregation, multi-branch depth-wise strip convolutions for capturing multi-scale context, and 1 × 1 convolutions for modeling inter-channel relationships. The output of the 1 × 1 convolution serves as an attention weight to reweight the input of the MSCA module. The core computation of MSCA is formulated as follows:

(1) $$\mathrm{Att}=Conv_{1\times 1}(\sum_{i=0}^{3}Scale_{i}(\mathrm{DW}-Conv(F)))$$

(2) Out=Att⊗F

**Figure 4:**
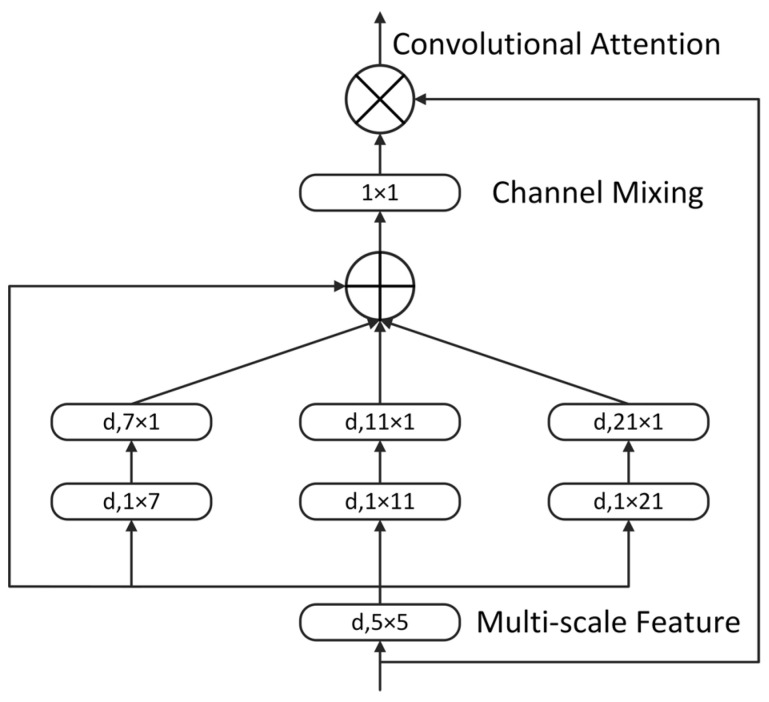
MSCA module.

In this study, the MSCA module was integrated into the Neck section of YOLO11, specifically after the C3K2 module, to enhance the model’s capability in capturing multi-scale contextual information, thereby improving overall detection performance. The MSCA attention module structure is illustrated in Figure 4.

#### 2.2.3. PPA Module

**Figure 5:**
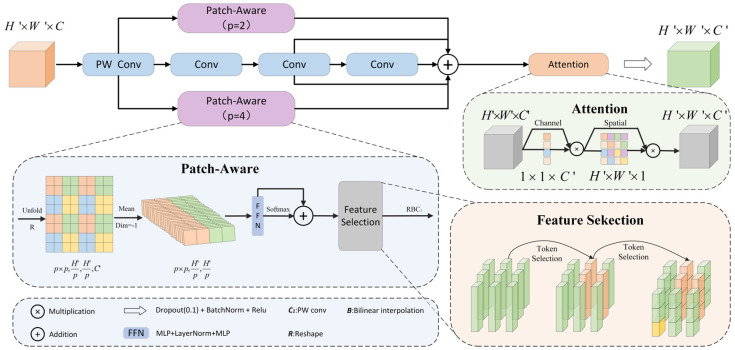
PPA module.

The Parallelized Patch-wise Attention (PPA) module [37] primarily addresses the issue of small object information loss caused by multiple down-sampling operations in detection networks. The PPA module consists of two key components: multi-branch fusion and an attention mechanism. It employs a parallel multi-branch approach comprising local, global, and serial convolutions, allowing the extraction of multi-scale and hierarchical features. This design enhances the ability to capture small object features, thus improving detection accuracy. The PPA attention module structure is depicted in Figure 5.

In this study, the PPA module was incorporated after the down-sampling operation and before the detection head in YOLO11, facilitating the capture of multi-scale features, efficient feature aggregation, and improved feature representation. This modification enhances the internal information flow within the model, strengthening its capability to extract features at various scales and hierarchical levels, ultimately improving the accuracy of small object eye detection.

#### 2.2.4. GAM Module

The Global Attention Mechanism (GAM) [38] aims to address limitations in traditional attention mechanisms, particularly in preserving information across channel and spatial dimensions. GAM adopts a sequential channel-spatial attention mechanism to overcome these challenges. The implementation details of GAM include a two-layer multilayer perceptron (MLP) to enhance cross-dimensional channel-spatial dependencies, improving the model’s ability to learn complex features. Additionally, two convolutional layers are used to fuse spatial information, enhancing spatial feature learning while avoiding max-pooling operations that may cause information loss. Finally, grouped convolutions and channel shuffling are employed to reduce computational overhead and memory usage. Through these design optimizations, GAM effectively captures global information and significantly improves model performance while maintaining high efficiency.

**Figure 6:**
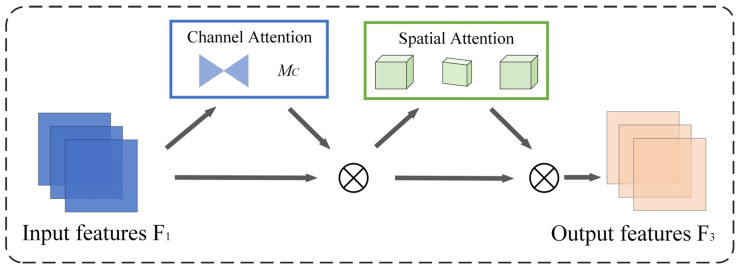
GAM module.

By embedding GAM into the final stage of the backbone network in YOLO11, specifically after the C2PSA module, the model benefits from GAM’s capability to learn complex features and integrate global information, thereby enhancing its ability to recognize different estrus stages. The structure of the GAM module is shown in Figure 6.

#### 2.2.5. ATFL Focus Loss Function

Adaptive Threshold Focal Loss (ATFL) [39] is designed to address class imbalance and improve the model’s focus on hard-to-classify samples, thereby enhancing detection performance. Inspired by the original Focal Loss, ATFL introduces adaptive weighting, a focal mechanism, threshold adjustment, and enhanced learning capacity. First, ATFL dynamically adjusts loss weights based on each sample’s characteristics and the model’s output, adapting the threshold according to the difference between predictions and ground-truth labels. This enables the model to concentrate more on hard-to-classify samples during training. Additionally, ATFL incorporates an adaptive threshold to compute an optimal value for each sample, determining how losses are weighted. This approach ensures that the loss function better reflects sample importance. By integrating a focal mechanism, ATFL reduces the influence of easy-to-classify samples while reinforcing attention on difficult samples. Lastly, ATFL optimizes loss computation to accelerate the learning of valuable features, thereby improving training efficiency and effectiveness.

The Threshold Focal Loss (TFL) function mitigates the impact of easy samples by reducing their loss weight while increasing the weight assigned to hard samples. The TFL formulation is as follows:

(3) $$\mathrm{TFL}=\left\{\begin{array}{l} -{\left(\lambda -P_{t}\right)}^{\eta }\log(P_{t}),P_{t}<=0.5\\ -{\left(1-P_{t}\right)}^{\gamma }\log(P_{t}),P_{t}>0.5 \end{array}\right.$$

where *η* and *λ* (*λ* > 1) are hyperparameters. For different datasets and models, these hyperparameters can be fine-tuned through multiple adjustments to achieve optimal performance. To enhance adaptability, modifications were made to *η* and *γ*. In general, higher probability values correspond to lower information gain, whereas lower probability values indicate higher information gain. Therefore, the adaptive modulation factor *γ* is formulated as

(4) $$\gamma =-\ln({\overset{\wedge }{P}}_{c})$$

During the later stages of network training, an excessively high expected probability value reduces the proportion of hard samples. Thus, *η* is defined as

(5) $$\eta =-\ln(P_{t})$$

By integrating the aforementioned components, the Adaptive Threshold Focal Loss (ATFL) is formulated. In this study, ATFL replaces the cross-entropy loss in YOLOv11 to enhance model performance. The introduction of ATFL enables YOLOv11 to focus more on hard-to-detect samples during training, thereby improving overall detection accuracy. Furthermore, the adaptive nature of ATFL allows the loss function to adjust automatically based on data distribution, enhancing the model’s generalization ability across diverse scenarios.

**Figure 7:**
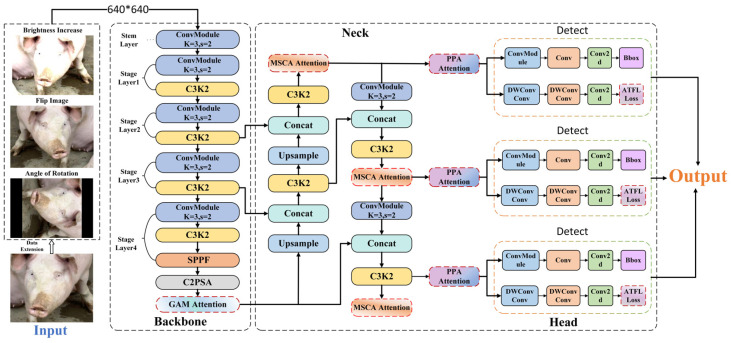
ECA–YOLO architecture.

The overall network architecture of the ECA–YOLO model is illustrated in Figure 7.

### 2.3. Experimental Platforms

This study was conducted on a Windows-based laptop equipped with an Intel Core i5-12450H @2.00 GHz processor and an NVIDIA GeForce 3060 Laptop GPU. The training and inference processes were implemented using the PyTorch (version 1.8.0) framework within the PyCharm integrated development environment, with Python 3.8 as the programming language. All comparative algorithms were executed under identical experimental conditions to ensure fairness. The core hardware and software configuration of this study is shown in Table 2.

### 2.4. Assessment Indicators

A confusion matrix [40] is a tabular representation used in machine learning and deep learning to evaluate the performance of classification algorithms. It provides a visual and analytical assessment of a model’s predictive results. The evaluation metrics used in this study are as follows:

(1) Accuracy

Accuracy represents the proportion of correctly predicted samples (both positive and negative) out of the total samples. Its mathematical formulation is given by Equation (6).

(6) $$\mathrm{Accuracy}=\frac{\mathrm{TP}+\mathrm{TN}}{\mathrm{TP}+\mathrm{FN}+\mathrm{TN}+\mathrm{FP}}$$

(2) Precision

Precision measures the proportion of correctly predicted positive samples among all predicted positive samples. The calculation is defined in Equation (7).

(7) $$\mathrm{Precision}=\frac{\mathrm{TP}}{\mathrm{TP}+\mathrm{FP}}$$

(3) Recall

Also known as sensitivity, recall quantifies the proportion of correctly predicted positive samples among all actual positive samples. Its computation is outlined in Equation (8).

(8) $$\mathrm{Recall}=\frac{\mathrm{TP}}{\mathrm{TP}+\mathrm{FN}}$$

(4) F1-Score

Since different models exhibit varying trade-offs between precision and recall, the F1-Score is used as a harmonic mean of both metrics to provide a balanced evaluation. The F1-Score computation is described in Equation (9).

(9) $$\text{F1-Score}=\frac{1}{\frac{1}{2}(\frac{1}{\mathrm{Precision}}+\frac{1}{\mathrm{Recall}})}$$

(5) GFLOPs

GigaFLOPs (GFLOPs) represent the number of floating-point operations (FLOPs) a model performs in billions. A lower GFLOP count typically indicates faster inference speed, reduced hardware requirements, and lower energy consumption.

(6) Parameters

Parameters refer to the total number of trainable weights and biases within a deep learning model. A higher parameter count enables the model to learn more complex features but also increases computational cost and memory usage. Large models generally require more data and longer training times to achieve good generalization while avoiding overfitting. In resource-constrained environments, balancing model accuracy, computational efficiency, and deployment feasibility makes parameter count a critical consideration.

(7) Detect Speed

In deep learning-based image classification tasks, Frames Per Second (FPS) refers to the number of image frames processed per unit time, serving as a key metric for evaluating inference speed and efficiency. It is computed as follows:

(10) $$\mathrm{FPS}=\frac{N}{T_{\mathrm{total}\;\mathrm{time}}}$$

where *N* represents the total number of detected frames, and *T*_total time_ denotes the total detection time (in seconds). Under standard conditions, the model inference speed is defined as 1000 ms/FPS. To better visualize the subtle impact of each improved module on overall speed in ablation experiments and to reflect the model’s sensitivity to short-term performance changes, we define the Detect Speed as follows in this study:

(11) $$\mathrm{Detect}\;\mathrm{speed}=\frac{500\;\mathrm{ms}}{\mathrm{FPS}}(\mathrm{ms}\cdot {\mathrm{frame}}^{-1})$$

As defined in Equation (11), the unit of Detect speed remains ms·frame^−1^. A lower Detect Speed indicates faster detection and higher computational efficiency.

## 3. Results

The main experimental parameter settings in this study are as follows: the input image size is 640 × 640 pixels, the initial learning rate (LR) is set to 0.01, the weight decay coefficient is 0.0005, and the momentum factor is 0.937. The number of training epochs is set to 200, with a batch size of 8 and num_workers set to 1. The optimizer used is Stochastic Gradient Descent (SGD). All results, including those from comparison algorithms, were obtained under identical hardware and software environments, with consistent parameter settings.

### 3.1. Ablation Experiment

To investigate the impact of different enhancement modules on model performance, an ablation study was conducted by integrating the proposed four improvements with YOLOv11 in various configurations. The models were trained, validated, and evaluated on the estrus dataset, and the final ablation study results are summarized in Table 3.

The ablation study in Table 3, using YOLOv11n as the baseline, demonstrates that introducing individual improvement modules enhances model performance to varying degrees. Among them, the PPA module achieves the most significant improvement, increasing mAP to 0.915 and F1-score to 0.860, followed by MSCA, GAM, and ATFL. However, considering detection speed, GAM and ATFL are more lightweight modules that improve mAP without imposing a significant computational burden, resulting in faster inference speeds. In contrast, PPA and MSCA incur slightly higher computational costs.

Regarding the effect of combined improvements, the PPA + GAM + ATFL combination achieves an mAP of 0.925 and an F1-score of 0.870, with the number of parameters increasing only to 5.20M. This suggests that the synergy between attention mechanisms and an optimized loss function effectively enhances feature representation. The MSCA + PPA + ATFL combination further improves multi-scale expression feature adaptability, reaching an mAP of 0.928 and an F1-score of 0.870. Meanwhile, the MSCA + PPA + GAM combination, despite lacking loss function optimization, attains an mAP of 0.930, highlighting the significant contribution of attention modules in fine-grained feature recognition. When all modules are integrated, the model achieves its best recognition performance, with an mAP of 0.932 and an F1-score of 0.880, at the cost of a minor decrease in detection speed (Detect Speed increased by 0.42).

**Figure 8:**
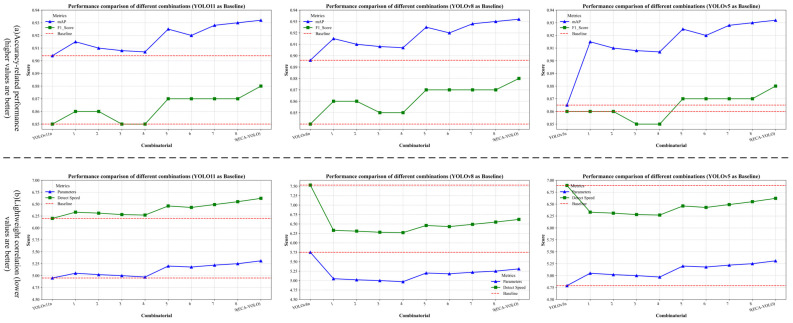
Ablation experiment.

Overall, compared to the original model, ECA–YOLO achieves substantial performance gains with a minimal increase in parameter count, making it well-suited for sow estrus detection scenarios that require high-precision detection. Figure 8 illustrates the improvements achieved by different combinations of enhancements based on YOLOv11n, YOLOv8n, and YOLOv5n as baselines.

### 3.2. ECA–YOLO Training Results

**Figure 9:**
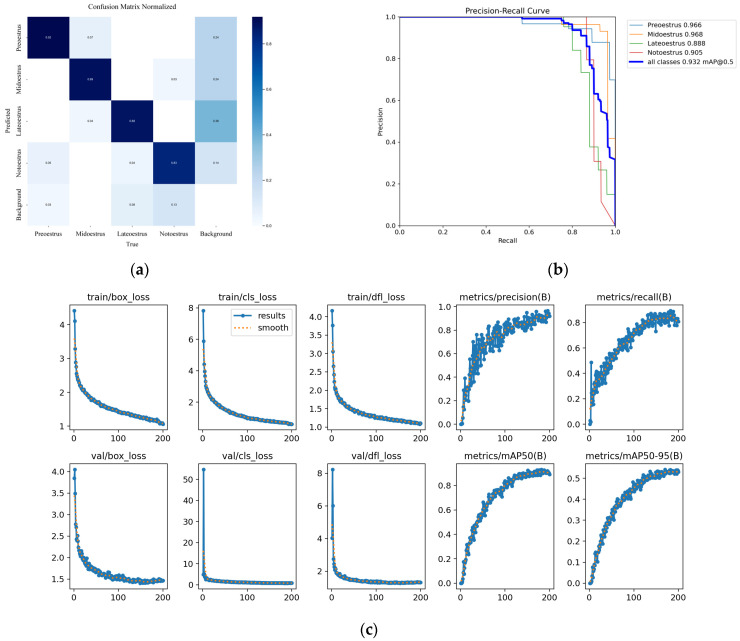
ECA–YOLO experiment results. (**a**) Confusion matrix normalization, (**b**) P–R curve, (**c**) Curve changes during training and validation.

The normalized confusion matrix of ECA–YOLO during iterations on the estrus eye dataset is presented in Figure 9a, while the precision–recall (P–R) curve is shown in Figure 9b. The training and validation loss curves are illustrated in Figure 9c.

### 3.3. Comparison of Similar Models

**Figure 10:**
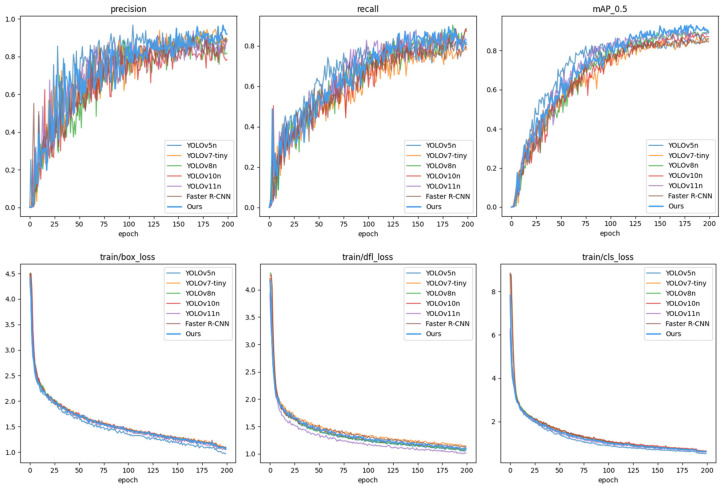
ECA–YOLO experiment results.

To further evaluate the performance of ECA–YOLO, comparative experiments were conducted on the estrus eye dataset against YOLOv5, YOLOv7Tiny, YOLOv8, YOLOv10, and Faster R-CNN. The iterative training curves are depicted in Figure 10, with detailed experimental results listed in Table 4.

**Figure 11:**
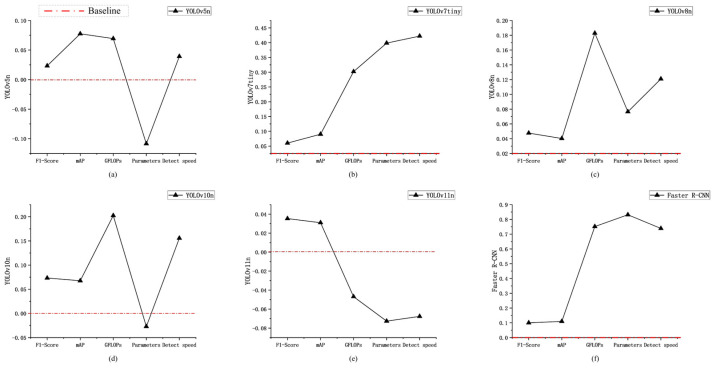
Performance improvement rate of ECA–YOLO over other participating algorithms in each comparison. (**a**) YOLOv5n, (**b**) YOLOv7-tiny, (**c**) YOLOv8n, (**d**) YOLOv10n, (**e**) YOLOv11n, (**f**) Faster R-CNN.

ECA–YOLO achieves an mAP of 93.2% and an F1-score of 88.0% on the estrus eye dataset. Compared to YOLOv5n, YOLOv7Tiny, YOLOv8n, YOLOv10n, YOLOv11n, and Faster R-CNN, its mAP improves by 7.74%, 9.00%, 4.01%, 6.75%, 3.09%, and 10.95%, respectively. The GFLOPs, parameter count, and detect speed of ECA–YOLO are 6.7, 5.31M, and 6.62 (according to the custom Equation (11) defined in this study, the conversion to standard FPS is approximately 75.53 frames per second.), respectively. While the parameter count increases by 10.85% compared to YOLOv5n, the F1-score and mAP improve by 2.32% and 7.74%, respectively, while the computational cost (GFLOPs) decreases by 6.94%. Compared to YOLOv8n, which also performs well, ECA–YOLO achieves superior F1-score and mAP while significantly optimizing GFLOPs, parameter count, and detection speed. Compared to the original YOLOv11n, ECA–YOLO exhibits a trade-off: GFLOPs, parameter count, and detection speed decrease by 4.68%, 7.27%, and 6.77%, respectively, but F1-score and mAP improve by 3.52% and 3.09%. The relative performance improvements of ECA–YOLO compared to other algorithms are visualized in Figure 10. Detailed recognition results per category are summarized in Table 5, while category-wise visualization results are shown in Figure 11.

**Figure 12:**
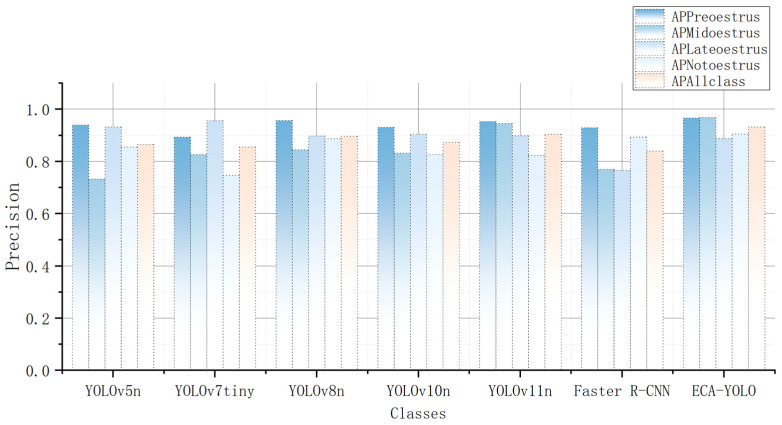
Comparison of recognition results of each algorithm on each class.

Analyzing all the results, ECA–YOLO achieves a considerable improvement in detection accuracy over YOLOv11n, with only a slight increase in computational cost, parameter count, and detection time. When using the smallest versions of YOLOv5, YOLOv8, and YOLOv10 as baseline models, ECA–YOLO maintains a clear advantage in terms of detection accuracy, model size, and detection speed, aligning well with the intended lightweight design. Detailed recognition results for each category are presented in Table 6, with visualized results shown in Figure 12.

The results indicate that ECA–YOLO achieves the best recognition performance for early estrus, mid-estrus, and non-estrus stages, surpassing the six other comparative algorithms. Specifically, ECA–YOLO attains an average precision of 96.6% for early estrus detection, outperforming YOLOv5n, YOLOv7tiny, YOLOv8n, YOLOv10n, YOLOv11n, and Faster R-CNN by 2.87%, 8.17%, 0.94%, 3.75%, 1.36%, and 3.98%, respectively. For mid-estrus detection, ECA–YOLO achieves an average precision of 96.8%, improving upon the aforementioned models by 32.24%, 17.19%, 14.69%, 16.34%, 2.43%, and 25.71%, respectively. Moreover, its recognition accuracy for non-estrus reaches 90.50%, representing enhancements of 5.84%, 21.15%, 2.02%, 9.56%, 10.09%, and 1.34% over the comparative models. Regarding late estrus detection, ECA–YOLO attains an average precision of 88.80%, slightly lower than YOLOv5n, YOLOv7tiny, YOLOv8n, YOLOv10n, and YOLOv11n by 4.72%, 7.11%, 1.00%, 1.76%, and 1.11%, respectively. However, its mean average precision (mAP) surpasses these models by 7.74%, 9.00%, 4.01%, 6.75%, and 3.09%, respectively.

From an overall perspective, YOLOv5n, YOLOv7tiny, and Faster R-CNN exhibit highly imbalanced performance across the four estrus categories, with an average precision disparity of 20.70%, 20.90%, and 16.30%, respectively, between the highest and lowest performing categories. This indicates that these models lack sufficient sensitivity to certain estrus-related ocular feature variations, leading to substantial discrepancies in classification accuracy. In contrast, YOLOv8n, YOLOv10n, and YOLOv11n demonstrate more balanced recognition performance, with precision disparities of 11.30%, 10.50%, and 13.10%, respectively. The improved YOLOv11n achieves the most balanced recognition performance with the highest overall precision, demonstrating that the incorporation of various modules enhances feature attention and attention mechanisms, enabling the model to comprehensively capture and learn features across different estrus stages. Additionally, the ATFL module further mitigates the negative effects of class imbalance, ultimately achieving more stable and efficient recognition performance.

**Figure 13:**
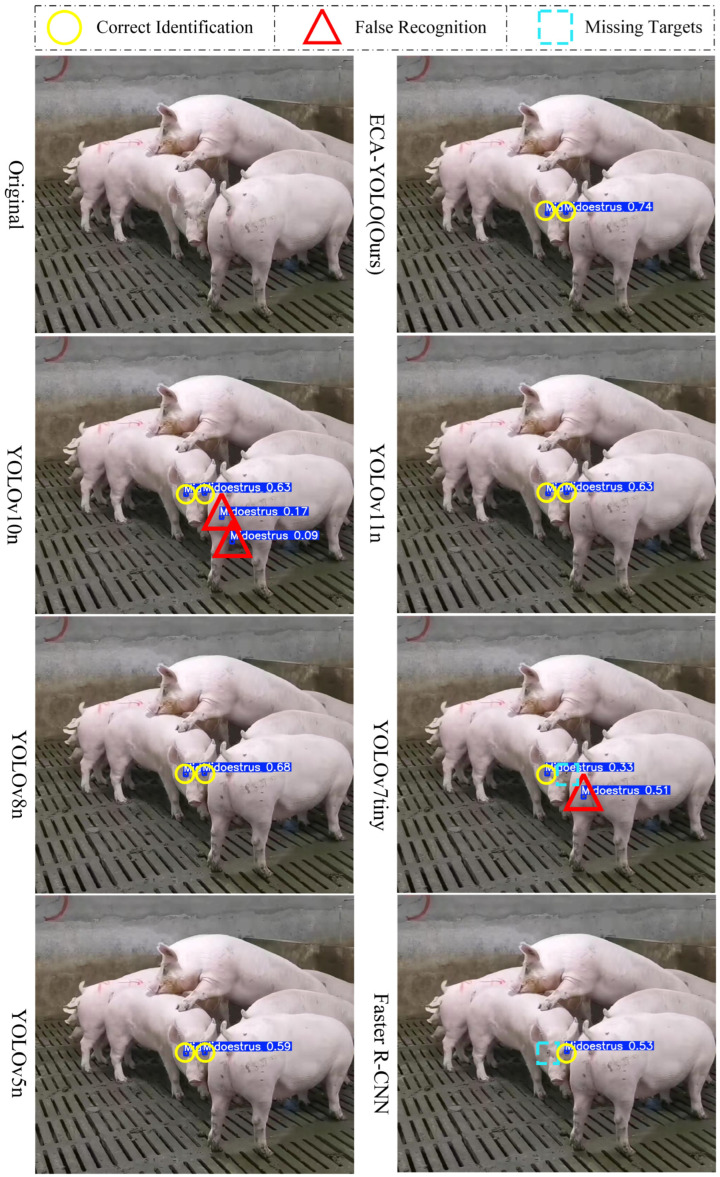
Recognition Performance in Complex Environments.

Figure 13 presents the detection results of various models under complex pig house environments, where multiple target pigs overlap with intricate backgrounds (boars engaging in mounting behavior are marked in the images). It is evident that YOLOv10 and YOLOv7tiny exhibit false detections, erroneously classifying other environmental objects as mid-estrus eyes. Furthermore, Faster R-CNN and YOLOv7tiny suffer from missed detections. In contrast, ECA–YOLO not only accurately identifies both eyes of sows in mid-estrus but also achieves high confidence scores. Notably, the baseline models YOLOv11n, YOLOv8n, and YOLOv5n also perform relatively well.

## 4. Model Verification and Discussion

### 4.1. Model Validation

#### 4.1.1. Validation Data Collection

To validate the effectiveness and reliability of the proposed ECA–YOLO model and ensure it does not overfit the training dataset, the research team conducted experiments at the Fuguo Ecological Breeding Cooperative in Pianguan County, Xinzhou City, Shanxi Province, China (longitude 111.507996, latitude 39.436196). A preliminary sow estrus warning system was also established. The experiment involved 10 primiparous sows, none of which were included in the model training, all housed in individual gestation stalls. This setup ensured data independence and scientific validity of the results. As shown in Figure 1b, high-definition cameras were installed above the stalls, and the collected data were transmitted in real-time via a smart gateway to a monitoring room for further analysis and evaluation.

For validation, serum hormone analysis was conducted, using fluctuations in estradiol (E2), progesterone (P4), and luteinizing hormone (LH) levels as reference indicators to assess the accuracy of the system’s predictions.

#### 4.1.2. Validation Experiment Procedure

(1) Eye Image Collection: High-definition cameras were installed in the trial pig farm following the setup in Figure 1 to capture eye images of sows at different estrus stages. The collected data were transmitted in real time to the monitoring room via an intelligent gateway.

(2) Estrus Warning System Determination: Based on the acquired eye image data, the estrus warning system automatically determines whether a sow is in estrus and further classifies the estrus stage (non-estrus, early estrus, mid-estrus, or late estrus) through feature analysis.

(3) Serum Hormone Analysis: When the system predicted that a sow was in a specific estrous stage, immediate serum sampling was performed to measure the concentrations of E2, P4, and LH. These hormonal fluctuations are key physiological markers of estrus and provided an effective means to verify the accuracy of the estrus warning system.

The final validation results obtained are shown in Table 7.

As shown in Table 6, the model achieved a precision of 91.16%, a recall of 90.20%, and an F1-score of 90.67%, demonstrating strong practical performance. These results indicate that ECA–YOLO does not overfit the estrus sow eye dataset used for model training and suggest its potential applicability in real-world pig farms.

### 4.2. Discussion

#### 4.2.1. Analysis of Model Limitations

**Figure 14:**
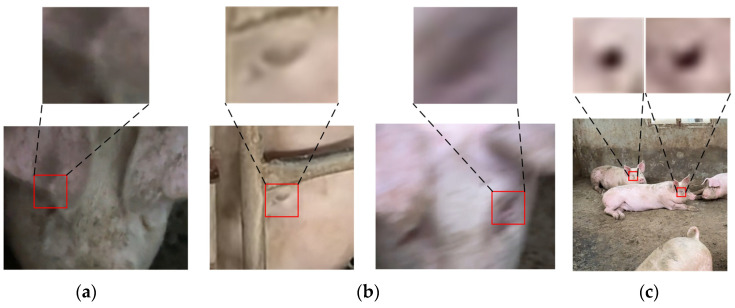
Several scenarios that impose limitations on the model. (**a**) Ear self-occlusion, (**b**) Visual angle problem, (**c**) Motion blur.

Despite the incorporation of the MSCA module to enhance the detection capability for small ocular targets in this study, detection accuracy may decline in more complex or severely occluded environments. Under conditions such as uneven lighting or partial occlusion of the sow’s head, model accuracy may be affected, resulting in detection failures or misclassifications. As shown in Figure 14, failure cases arise due to self-occlusion by ears, viewpoint constraints, motion blur, and excessively small target sizes (where the eye region’s pixel resolution is too low to discern appearance changes). In such extreme scenarios, model performance is inherently constrained.

In this study, all sows used for image data collection and model training belonged to the Large White breed. It is noteworthy that factors such as breed, age, and exposure to boars can influence estrous cycle duration, estrus period length, and ocular features, potentially affecting model performance to varying degrees. Further experimental validation and practical analysis are required to assess these influences. Additionally, artificial insemination remains an essential step in practical applications. Since ECA–YOLO relies solely on image-based detection, the identified estrus stage may be slightly earlier or later than human judgment. Therefore, integrating automated detection results with manual assessments is necessary to optimize conception rates.

Despite its strong performance in estrus detection, several challenges hinder ECA–YOLO’s adoption in commercial pig farming. One primary limitation is the initial investment cost. Although ECA–YOLO benefits from a relatively low hardware deployment requirement (as standard cameras suffice for image acquisition), deep learning-based detection systems still require dedicated computing infrastructure, such as servers and network equipment, which may be cost-prohibitive for small- and medium-sized farms. Additionally, system maintenance and calibration are critical for ensuring long-term reliability, as environmental factors like dust and lighting variations can affect detection performance. Another key challenge is technological acceptance. Traditional methods, such as the back-pressure test, remain widely used due to their simplicity and proven effectiveness, making farmers reluctant to adopt AI-assisted detection. Experienced stockmen may perceive limited added value, whereas large-scale automated farms are more likely to benefit from ECA–YOLO’s efficiency in reducing labor-intensive monitoring. Addressing these challenges through cost-effective implementation strategies, user-friendly system design, and integration with existing breeding management protocols is essential for promoting ECA–YOLO’s practical application in pig farms.

#### 4.2.2. Discussion of the Impact of Confidence Thresholds on ICAE-YOLO Performance

**Figure 15:**
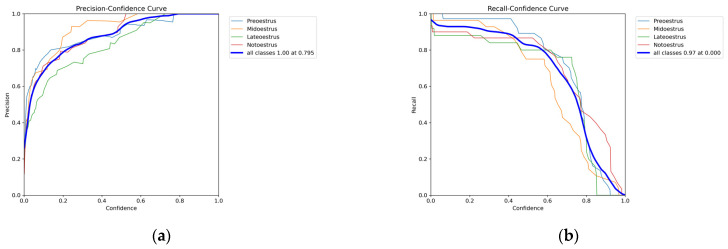
P-curve and R-curve. (**a**) P-curve, (**b**) R-curve.

Balancing confidence threshold selection and detection efficiency is critical in object detection algorithms, as the confidence threshold directly influences model performance. A comprehensive analysis of Figure 15a,b reveals that, as the confidence threshold increases, precision continually improves, reaching its peak (1.0) at 0.795, indicating more reliable predictions and fewer false positives. Conversely, recall declines as the confidence threshold increases, reaching a maximum of 0.97 at 0 and rapidly decreasing beyond 0.6, suggesting a significant increase in missed detections. These findings indicate that the confidence threshold can serve as a performance indicator in practical estrus detection. Lower confidence thresholds can be selected to minimize missed detections, while higher thresholds can be used to reduce false positives. The threshold selection can be adjusted based on specific application requirements to optimize detection outcomes.

## 5. Conclusions

Building upon YOLOv11, this study develops an enhanced automatic estrus detection model, ECA–YOLO. By leveraging the structural similarity between pig and human eyes, the model extracts ocular appearance features across different estrus stages to achieve precise classification into pre-estrus, estrus, post-estrus, and non-estrus phases. While ensuring high detection performance, the model remains lightweight, meeting the requirements for real-time, all-weather monitoring of sow estrus. The proposed method is applicable to large-scale pig farms, enabling non-contact estrus detection. The key findings of this study are as follows:

(1) The study investigates ocular appearance variations across different estrus stages and establishes a dataset of sow eye images covering pre-estrus, estrus, and post-estrus periods. Validation results show that ECA–YOLO achieves a mean average precision (mAP) of 93.2%, an F1-score of 88.0%, with model parameters of 5.31M, and FPS reaches 75.53 frames per second.
(2) Experimental results indicate significant phenotypic changes in the eye region across estrus stages, confirming that ocular features can serve as reliable indicators for estrus detection.
(3) Compared to YOLOv5n, YOLOv7tiny, YOLOv8n, YOLOv10n, YOLOv11n, and Faster R-CNN, ECA–YOLO achieves the highest detection accuracy while maintaining a fast inference speed.

In the future, our team will further explore the estrus differences among sows caused by factors such as breed, parity, and age, aiming to reveal how these factors influence the phenotypic changes in the eyes of sows before and after estrus. Meanwhile, our model will be further enhanced to achieve greater robustness and faster processing speed. In addition, our method will be validated in more pilot pig farms.

## Figures and Tables

**Table 1:** Distribution of the ocular dataset of sows in estrus.

Category	Number of Raw Data	Total Number of Raw Data	Expansion Method	Number of Expanded Data	Total Number of Expanded Data Sets
Non-estrus	382	1487	1.Brightness Increase 2.Flip Image 3.Angle of Rotation	1146	4461
Pre-estrus	369			1107	
Mid-estrus	371			1113	
Late-estrus	365			1095	

**Table 2:** Core configuration and model parameter settings.

**Core Hardware** **Configuration**	Processor	Intel Core i5-12450H
	Graphics Card	NVIDIA GeForce 3060 Laptop
	Memory	16G
	Hard Disk	512G SSD
**Core Software** **Configuration**	Anaconda	Anaconda3 2019.10(64-bit)
	Python	3.8
	CUDA	11.2
	torch	1.8.0
	TorchVision	0.9.0
	Labelimg	1.8.6
	Operating System	Windows11(24H2)

**Table 3:** Ablation experiment results.

Model	mAP	F1-Score	Parameters (M)	Detect Speed (ms·Frame^−1^)
YOLOv11n(baseline)	0.904	0.850	**4.95**	**6.20**
YOLOv11n + PPA	0.915	0.860	5.05	6.33
YOLOv11n + MSCA	0.910	0.860	5.02	6.31
YOLOv11n + GAM	0.908	0.850	5.00	6.28
YOLOv11n + ATFL	0.907	0.850	4.97	6.27
YOLOv11n + PPA + GAM + ATFL	0.925	0.870	5.20	6.46
YOLOv11n + MSCA + GAM + ATFL	0.920	0.870	5.18	6.43
YOLOv11n + MSAC + PPA + ATFL	0.928	0.870	5.22	6.49
YOLOv11n + MSCA + PPA + GAM	0.930	0.870	5.25	6.55
YOLOv11n + MSCA + PPA + GAM + ATFL(ECA-YOLO)	**0.932**	**0.880**	5.31	6.62

**Table 4:** Detailed data of comparison experiments.

Models	mAP	F1-Score	GFLOPs	Parameters (M)	Detect Speed (ms·Frame^−1^)
YOLOv5n	0.865	0.86	7.2	**4.79**	6.89
YOLOv7tiny	0.855	0.83	9.6	8.83	11.46
YOLOv8n	0.896	0.84	8.2	5.75	7.53
YOLOv10n	0.873	0.82	8.4	5.17	7.84
YOLOv11n	0.904	0.85	**6.4**	4.95	**6.20**
Faster R-CNN	0.840	0.80	27	31.49	25.35
ECA–YOLO	**0.932**	**0.88**	6.7	5.31	6.62

**Table 5:** Various performance improvement rates of ECA–YOLO over other algorithms.

Models	mAP Boost Rate (%)	F1-Score Boost Rate (%)	GFLOPs Reduction Rate (%)	Parameters Reduction Rate (%)	Detect Speed Reduction Rate (%)
YOLOv5n	7.7457	2.3256	6.9444	−10.8559	3.9187
YOLOv7tiny	9.0058	6.0241	30.2083	39.8641	42.2339
YOLOv8n	4.0179	4.7619	18.2927	7.6522	12.0850
YOLOv10n	6.7583	7.3171	20.2381	−2.7079	15.5612
YOLOv11n	3.0973	3.5294	−4.6875	−7.2727	−6.7742
Faster R-CNN	**10.9524**	**10.0000**	**75.1852**	**83.1375**	**73.8856**

**Table 6:** Recognition results of each algorithm on each class.

	AP_Pre-estrus_	AP_Mid-estrus_	AP_Late-estrus_	AP_Not-estrus_	AP_Allclass_
YOLOv5n	0.939	0.732	0.932	0.855	0.865
YOLOv7tiny	0.893	0.826	**0.956**	0.747	0.855
YOLOv8n	0.957	0.844	0.897	0.887	0.896
YOLOv10n	0.931	0.832	0.904	0.826	0.873
YOLOv11n	0.953	0.945	0.898	0.822	0.904
FasterR-CNN	0.929	0.770	0.766	0.893	0.840
ECA–YOLO	**0.966**	**0.968**	0.888	**0.905**	**0.932**

**Table 7:** Model scores.

Indicator	Value (%)
Precision	91.16
Recall	90.20
F1-Score	90.67

## Data Availability

The authors do not have permission to share data.
